# ECT: A Rapid and Sustained Response in Treatment-Resistant Psychotic Depression in the Elderly

**DOI:** 10.1192/j.eurpsy.2025.1347

**Published:** 2025-08-26

**Authors:** O. Martin Santiago, B. Arribas-Simon, O. Segurado-Martin, P. Martinez-Gimeno, M.-J. Mateos-Sexmero, M. Rios-Vaquero

**Affiliations:** 1 Hospital Clinico Universitario de Valladolid, Valladolid, Spain

## Abstract

**Introduction:**

Electroconvulsive Therapy (ECT) is a proven treatment for treatment-resistant depression (TRD), especially in elderly patients. Managing depression in this population is challenging due to comorbidities and medication intolerance. Research suggests that factors like melancholic features and early symptom improvement predict a positive response to ECT. ECT offers rapid and sustained antidepressant effects.

**Objectives:**

To present the case of a 74-year-old woman with TRD who successfully underwent ECT after failing multiple medications.

**Methods:**

A literature review was conducted on ECT for TRD in elderly patients. The clinical case is detailed, focusing on treatment, ECT application, and outcomes.

**Results:**

The patient had a history of severe depressive episodes. Previous hospitalizations were managed with tricyclic antidepressants, lithium, and olanzapine. However, lithium was discontinued after discharge due to subclinical hypothyroidism and renal function impairment. Although the patient remained stable for a time, her mood progressively worsened, leading to a marked decline in daily functioning and eventual admission to the psychiatric unit. Upon admission, the patient presented with severe depression, including loss of functionality, self-neglect, and passive suicidal ideation, hyporeactive state, significant vegetative symptoms, and moderate-to-severe anxiety. Given the lack of response to a comprehensive pharmacological regimen, ECT was initiated. The patient underwent six sessions of ECT, with initial improvements observed after the first session. By the third session, she showed marked improvements in mood, energy, and anxiety levels. By the end of the ECT course, she had regained full functionality and emotional stability.

**Conclusions:**

This case underscores the effectiveness of ECT in managing psychotic depression in elderly patients when pharmacological treatments are ineffective or poorly tolerated. The patient’s rapid response aligns with previous findings suggesting that early symptom improvement predicts favorable ECT outcomes. Additionally, the presence of melancholic features may have contributed to the success of ECT, as described in the literature. Given the patient’s history of lithium intolerance and multiple pharmacological failures, ECT emerged as the most viable treatment option. ECT also demonstrated long-term benefits.

This case also highlights the importance of considering ECT earlier in the treatment process for elderly patients and demonstrates the crucial role of ECT in achieving rapid and sustained recovery in elderly patients with psychotic depression resistant to pharmacological treatments. Early intervention with ECT was essential for the patient’s full functional recovery, reinforcing its value as a therapeutic option in severe, treatment-resistant cases.

**Disclosure of Interest:**

None Declared

